# The Gut-Kidney Axis: Putative Interconnections Between Gastrointestinal and Renal Disorders

**DOI:** 10.3389/fendo.2018.00553

**Published:** 2018-09-19

**Authors:** Markku Lehto, Per-Henrik Groop

**Affiliations:** ^1^Folkhälsan Institute of Genetics, Folkhälsan Research Center, Helsinki, Finland; ^2^Abdominal Center of Nephrology, University of Helsinki and Helsinki University Hospital, Helsinki, Finland; ^3^Research Programs Unit, Diabetes and Obesity, University of Helsinki, Helsinki, Finland; ^4^Department of Diabetes, Central Clinical School, Monash University, Melbourne, VIC, Australia

**Keywords:** type 1 diabetes, diabetic nephropathy, chronic kidney disease, gut permeability, inflammatory bowel disease, gastrointestinal inflammation, contact activation, intestinal alkaline phosphatase

## Abstract

Diabetic kidney disease (DKD) is a devastating condition associated with increased morbidity and premature mortality. The etiology of DKD is still largely unknown. However, the risk of DKD development and progression is most likely modulated by a combination of genetic and environmental factors. Patients with autoimmune diseases, like type 1 diabetes, inflammatory bowel disease, and celiac disease, share some genetic background. Furthermore, gastrointestinal disorders are associated with an increased risk of kidney disease, although the true mechanisms have still to be elucidated. Therefore, the principal aim of this review is to evaluate the impact of disturbances in the gastrointestinal tract on the development of renal disorders.

## Introduction

Type 1 diabetes is an autoimmune disorder that is primarily associated with elevated blood glucose due to loss of the insulin-producing pancreatic beta-cells. Long disease duration and poor glycemic control increase the risk of micro- and macrovascular complications, and the incidence and severity of these complications is modulated by genetic and environmental factors. Diabetic nephropathy, affecting up to one third of individuals with type 1 diabetes, is a global health problem that is associated with significant and continuously growing health care costs (1, 2). Diabetic nephropathy is not only strongly associated with cardiovascular disease, but is also one of the leading risk factors for premature death in the Western countries (3–5).

The presence and progression of diabetic nephropathy is associated with low-grade chronic inflammation, which could also be seen as an indication of a systemic infection (6). It is evident that hyperglycemia by itself increases the risk of bacterial infections (7, 8), and particularly patients with poor glycemic control are susceptible to respiratory, urinary, skin, and intestinal infections (9–11). Moreover, severe bacterial infections increase the risk of acute kidney injury especially in patients with diabetes, in the elderly, and in those with renal impairment and hypertension (12). Dysbiosis of the gut microbiota has frequently been reported in various autoimmune and metabolic disease conditions such as in allergy, asthma, inflammatory bowel disease, celiac disease, systemic lupus erythematosus, arthritis, chronic kidney disease, diabetes, obesity, and cardiovascular disease (13–18). Based upon the hygiene hypothesis, frequent exposure to infective microbial agents in childhood protects from the development of autoimmune diseases later in life. Apparently, crosstalk between pathogenic and commensal gut bacteria play an important role in this process (19). In support of this hypothesis, certain viruses and bacteria have been linked to the development of autoimmunity (20–22).

**Figure 1:**
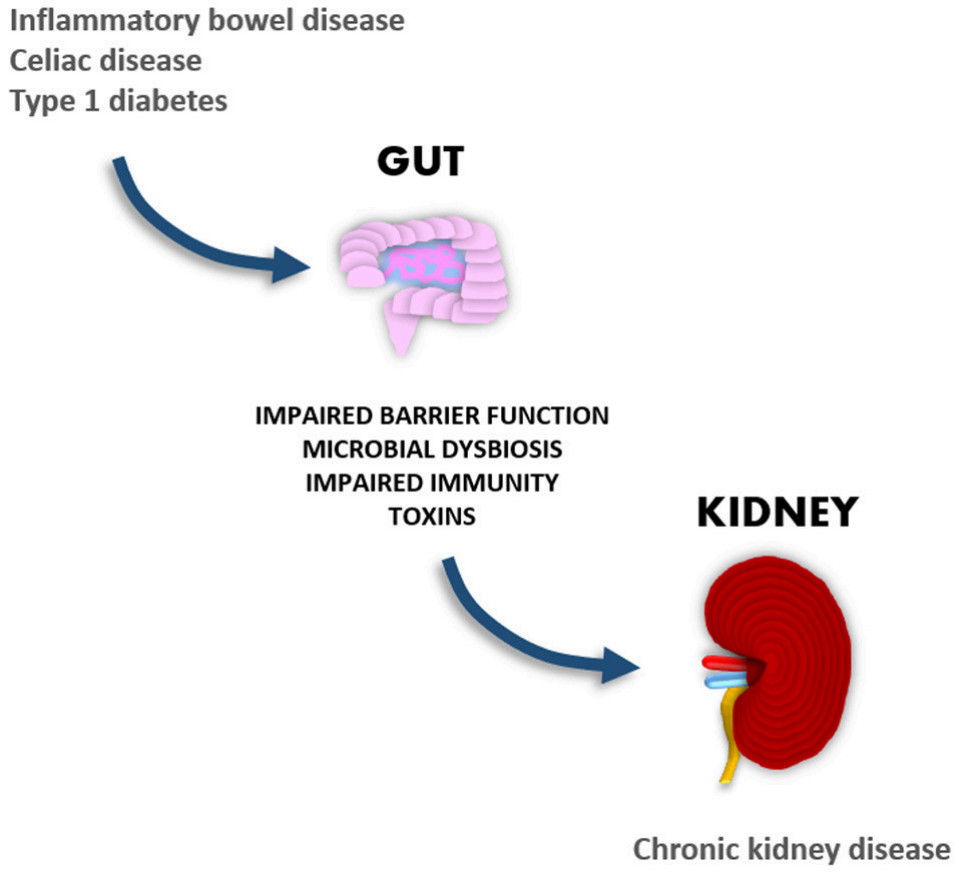
Gastrointestinal manifestations in autoimmune-mediated diseases are associated with the development of chronic kidney disease.

It is likely that gastrointestinal disorders associated with dysbiosis may play a significant role in the development of diabetic nephropathy, since disruption of the gastrointestinal wall increases the translocation of microbial compounds, which have been shown to have proinflammatory and nephrotoxic properties. In support of this theory, we and others have shown that elevated systemic levels of bacterial endotoxins are associated with the development and progression of renal disease (23–26). Given the large amount of emerging new data suggesting cross-talk between the gut and the kidneys, the aim of this review is to explore such interconnections and particularly the link between gastrointestinal and renal disorders (Figure 1).

## Gastrointestinal manifestations in patients with diabetes

In addition to the classical microvascular (retinopathy, nephropathy, neuropathy) and macrovascular complications (coronary artery disease, stroke, peripheral vascular disease), individuals with type 1 diabetes also display gastrointestinal problems. Although gastrointestinal-related complications are relatively common in individuals with diabetes, their recognition and management are often challenging. The most common gastrointestinal problems are esophageal dysmotility, gastro-esophageal reflux disease, gastroparesis, and enteropathy, and the severity of the problems often correlates with the presence of micro- and macrovascular complications. It is of note that life-style related factors such as composition of diet and exercise as well as medication (e.g., metformin, statins, incretin-based therapies) also have an impact on the gastrointestinal problems (27). Up to 75% of individuals with diabetes may experience gastrointestinal symptoms such as early satiety, postprandial fullness, nausea, abdominal pain, vomiting, bloating, diarrhea and/or constipation affecting their quality of life (28). Although many of these symptoms could be attributed to diabetes-related autonomic neuropathy, the etiology of these gastrointestinal problems often remains unclear. However, in individuals with diabetes, a relationship between cardiovascular autonomic neuropathy and small-bowel bacterial overgrowth has been reported (29, 30).

Exposure to antibiotics may lead to disruption of the normal microbiota, which eventually could lead to colonization of pathogenic microbes (e.g., *Helicobacter pylori, Clostridium difficile, Candida* etc,). In individuals with type 1 diabetes in the UK, the rate of gastrointestinal infections was ~2-fold higher compared to the general population (8). The frequent use of antibiotics, which often disrupts the balance of the normal intestinal microflora, has also been shown to correlate with the severity of diabetic nephropathy in patients with type 1 diabetes (7). However, chronic gastrointestinal symptoms could also arise from the presence of other systemic diseases such as celiac disease, lactose intolerance, irritable bowel syndrome, and inflammatory bowel disease. Type 1 diabetes and inflammatory bowel disease are multifactorial autoimmune diseases, which share many genetic and immunological aspects (31, 32). Both are chronic diseases that are associated with increased risk of cardiovascular disease and premature mortality (33, 34). Despite the potential overlaps, earlier studies have not been able to demonstrate a higher risk of diabetes in patients with inflammatory bowel disease (35–38). However, in a recent study from the UK, the prevalence of clinical inflammatory bowel disease in adult individuals with type 1 diabetes was ~6-fold higher compared to non-diabetic controls (1.5 vs. 0.3%, respectively) (39). Nevertheless, although gastrointestinal manifestations are frequently reported in individuals with diabetes, the potential impact of gastrointestinal-related disorders on the development and progression of diabetic nephropathy remains to be elucidated (40).

## Gastrointestinal manifestations in patients with renal diseases

Chronic kidney disease is a multifactorial disorder primarily associated with the gradual loss of kidney function over time. Based on a large systematic review and meta-analysis comprising nearly seven million adult patients, the global estimate of the prevalence was about 13% (41). Diabetes is one of the leading causes, and the prevalence of chronic kidney disease is about 5 times higher among individuals with diabetes than in the general population (42). Patients with advanced chronic kidney disease carry a significantly higher risk of premature mortality due to increased risk of infection related diseases (43, 44). Consequently, sepsis is a severe life-threatening condition especially in patients with impaired renal function (45, 46).

A key problem in chronic kidney disease is the gut dysbiosis and the accumulation of uremic toxins. These uremic toxins are derived from the diet, the protein metabolism, and the metabolic action of the gut bacteria. The most intensively studied toxins are p-cresylsulphate and indole sulfate. Tyrosine and phenylalanine are the main sources of p-cresol, while indole originates from tryptophan. These intermediate by-products are absorbed from the gut and finally sulfated by the liver. The accumulation of the uremic toxins has many adverse effects, and for instance the accumulation of urea increases the urea influx into the intestinal lumen, where it is hydrolyzed to ammonia by microbial urease. Consequently, ammonium hydroxide, a by-product of ammonia, increases the intestinal pH leading to mucosal irritation and structural damage. The uremic condition in combination with chronic inflammation and impaired renal function may increase the risk of kidney disease progression (47, 48).

Metabolic dysregulation seen in individuals with diabetes is exacerbated by microbial dysbiosis and associated with expansion of anaerobic bacteria, defects in the intestinal barrier function, and increased translocation of microbial compounds, e.g., bacterial endotoxins. Moreover, significant alterations of the gut microbiota have been reported in patients with advanced kidney disease (49, 50). Also, the immunosuppressive treatment in patients with kidney transplants may have adverse effects on the gastrointestinal tract (51). Previous studies in animals and humans have shown that therapies targeting the gut might provide novel tools for the correction of intestinal metabolism and microbial dysbiosis in chronic kidney disease (52). For instance, in collagen type 4α3–deficient mice with progressive chronic kidney disease, the eradication of anaerobic microbiota with antibiotics prevented bacterial translocation, reduced the serum endotoxin levels, and reversed the systemic inflammation to the level of non-uremic controls (53). Although the results from dietary interventions in humans are still somewhat controversial, modulation of the intestinal microbiota by supplementation with probiotics may have beneficial therapeutic effects in individuals with impaired renal function (54).

IgA nephropathy is the most common form of primary glomerulonephritis worldwide. The renal injury is mediated by deposition of IgA antibodies in the glomerular mesangium leading to increased local inflammation and renal dysfunction. Increased reactivity to dietary antigens has been associated with mucosal inflammation. Interestingly, a recent genome-wide association study on individuals with IgA nephropathy of European and East Asian ancestry identified several risk loci, which have previously been associated with the risk of inflammatory bowel disease, intestinal epithelial barrier function, and immune response to mucosal pathogens (55). Furthermore, increased intestinal permeability, typically seen in inflammatory bowel disease and celiac disease, has also been reported in patients with IgA nephropathy (56–58).

## Kidney manifestations in patients with gastrointestinal disease

The inflammatory bowel disease is a multifactorial disease primarily associated with microbial dysbiosis, intestinal epithelial damage, immune dysregulation, and inflammation of the gut. Despite the growing knowledge, the etiology and pathogenesis of this complex disease is not yet fully understood. The incidence of extra-intestinal manifestations (e.g., in the eye, skin, bone, liver, pancreas, kidney, lung, and the heart) and other autoimmune related diseases (e.g., celiac disease, type 1 diabetes, sarcoidosis, asthma, psoriasis, and rheumatoid arthritis) seem to be significantly higher in patients with inflammatory bowel disease compared to the general population (59, 60). It has been estimated that 4–23% of patients with inflammatory bowel disease suffer from reno-ureteral complications (61), of which the most common are nephrolithiasis, tubulointerstitial nephritis, glomerulonephritis, and amyloidosis. A significant proportion of these renal complications are, however, associated with the use of medication (e.g., aminosalisalicytes, cyclosporine, azathioprine, and tumor necrosis factor alpha inhibitors) (62). Based on the histopathologic examination of kidney biopsies, IgA nephropathy and tubulointerstitial nephritis were the most common findings observed in patients with inflammatory bowel disease with subsequent kidney disease (63). Notably, inflammatory bowel disease patients with active disease have shown higher urinary albumin levels compared to those in the remission phase (64, 65).

Celiac disease is an autoimmune disease mainly associated with defects in the small intestine. Patients with celiac disease suffer from diarrhea, malabsorption, weight loss, iron deficiency, and anemia. Celiac disease is primarily caused by an aberrant autoimmune reaction toward gluten, which is an abundant protein present in various grains e.g., wheat, oats, rye, and barley. The diagnosis of celiac disease requires specific serologic blood tests (tissue transglutaminase IgA, deaminated gliadin peptide IgA/IgG, endomysial IgA) and small intestinal biopsies. Abnormal villous structures, villous atrophy, crypt hyperplasia, and increased number of intraepithelial lymphocytes are common endoscopic findings in patients with active disease. The primary treatment of celiac disease is gluten-free diet, which results in improvements in many cases (66). In Europe, the prevalence of celiac disease in individuals with type 1 diabetes is about 6–8 times higher compared to the general population (67). Despite the increased risk of celiac disease, many patients with type 1 diabetes and celiac disease do not display gastrointestinal symptoms (68, 69). It has become evident that patients with type 1 diabetes and celiac disease share many genetic (e.g., HLA and non-HLA genes) and environmental (e.g., dietary antigens, viral infections) risk factors. Based on previous clinical surveys, celiac disease was also associated with other extra-intestinal manifestations such as endocrine, connective tissue, and pulmonary disorders (70, 71). Compared to the general population, patients with celiac disease carry a higher risk of renal diseases e.g., IgA nephropathy (72–75). Based on eight Northern European studies, the relative risk of kidney disease was ~2-fold higher in patients with celiac disease compared to those without. Subsequent subgroup analyses in patients with celiac disease showed ~1.5-fold and ~2.6-fold increased risks of diabetic nephropathy and IgA nephropathy, respectively (76).

## Potential novel therapeutic tools

### Probiotics

Probiotics are living non-pathogenic micro-organisms, the ingestion of which should provide health benefits to the host. Overall, probiotics are thought to have anti-inflammatory, anti-oxidative, and many other favorable gut-modulating properties (77). Especially bacterial species belonging to the genus of *Bifidobacterium and Lactobacillus*, support the humoral immune responses against environmental toxins and antigens. Over the past decade, the impact of supplementation with probiotics has been intensively studied in individuals with mild or severe gastrointestinal symptoms e.g., diarrhea, irritable bowel syndrome, and inflammatory bowel disease (78, 79). Probiotic treatment with butyrate-producing bacteria (e.g., *Faecalibacterium prausnitzii, Butyricicoccus pullicaecorum*) may improve epithelial barrier integrity and suppression of intestinal inflammation (80). Among the newly discovered gut residing bacteria, *Akkermansia muciniphila* has shown promise for the treatment of metabolic disorders (81). In patients with kidney disease, probiotic supplementation has also shown some beneficial effects on the glycemic control, uremic toxins, blood urea nitrogen, oxidative stress, and markers of inflammation (82–87). Similar trends have been observed in clinical trials using synbiotics, a combination of probiotics and prebiotics, which support the growth and activity of the beneficial micro-organisms in the gut (54, 88). In a mouse model of acute kidney injury, short-term probiotic pretreatment decreased the inflammation and protected the mice from the development of severe kidney damage (89).

### Resistant starch

Starches are considered prebiotics, which support the health and growth of the gastrointestinal microflora. Natural plant starches with high amylose content are resistant to digestion by intestinal amylases. Enzyme-resistant starches pass the upper intestinal tract and are finally fermented by the gut bacteria in the large intestine leading to increased production of beneficial metabolites. Many of the propitious effects (e.g., increased number of butyrate-producing bacteria, reduced cell proliferation, decreased inflammation, and cellular apoptosis) are thought to be mediated by an increased production of short-chain fatty acids including acetate, propionate, and butyrate. Clinical trials with resistant starches have shown some promising effects in the management of metabolic diseases including colon cancer, inflammatory bowel disease, obesity and diabetes (90). In a recent study in non-obese diabetic mice, resistant starch diet decreased the number of autoreactive T-cells and protected the mice against type 1 diabetes (91). In addition to improved glycemic control and insulin sensitivity (92), resistant starch containing diet could also exert renoprotective effects e.g., via the vitamin D metabolism (93). Dietary supplementation of digestion resistant starches has been shown to ameliorate the progression of kidney disease in rodents (94, 95). In hemodialysis patients, consumption of resistant starch for 6 weeks reduced the plasma levels of uremic toxins (96). Despite these promising effects, more studies are needed to evaluate the impact of dietary factors on the gut and the kidney function in patients with chronic kidney disease (97).

### Fecal transplantation

The basic principle of fecal microbiota transplantation is to transfer the normal microbiome from a healthy donor to individuals suffering from microbial dysbiosis. Isolated stool preparations can be transferred to the recipient with the aid of colonoscopy, an orogastric tube, enema, or by orally given capsules. The fecal microbiota transplantation strategy has successfully been applied in the treatment of infections caused by *Clostridium difficile* and especially in those patients that do not respond to the antibiotic treatment. It is of note that chronic *Clostridium difficile* infections increase the risk of colon-related complications such as megacolon and pseudomembraneous colitis. Alternative treatment options have become increasingly important as the prevalence of antibiotic resistant bacterial strains have exploded all around the world. The risk of *Clostridium difficile* infections is particularly high among the patients with inflammatory bowel disease—up to eight times higher risk than in matched controls (98). Also patients receiving immunosuppressive medication, e.g., patients with a kidney transplant, have a higher risk of colonization of pathogenic bacteria. In addition to *Clostridium difficile* infections, the fecal microbiota transplantation treatment has also been shown to be an effective tool for the eradication of gut residing *Salmonella* and ESBL-producing *E. coli* bacterial strains (99, 100). Besides chronic gastrointestinal infections, the fecal microbiota transplantation therapy may also offer novel treatment strategies for various metabolic diseases such as diabetes, obesity, non-alcoholic fatty liver disease, and inflammatory bowel disease (101, 102).

## Potential novel therapeutic targets

**Figure 2:**
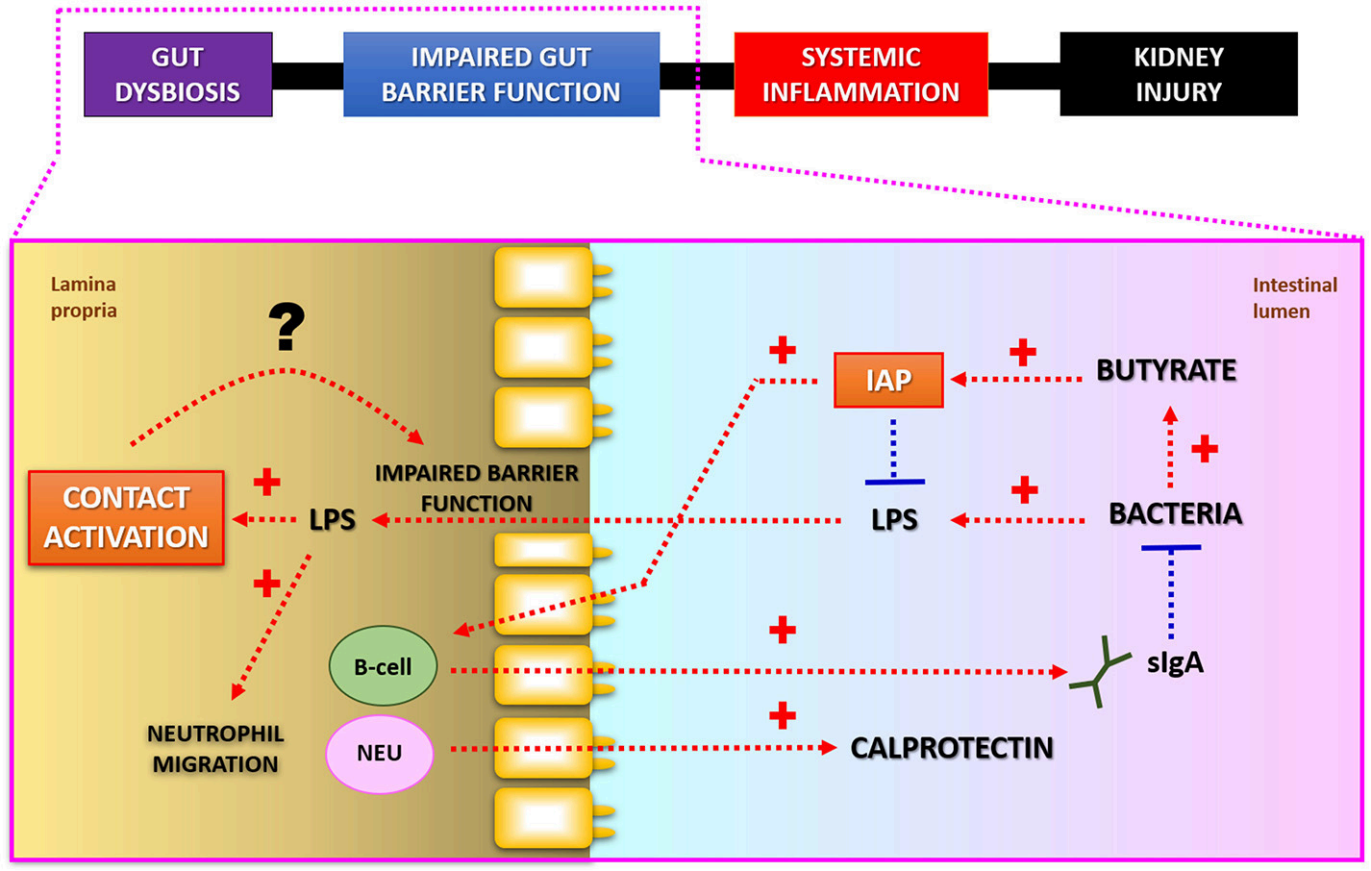
Putative interconnections between LPS-detoxification and intestinal barrier function. Intestinal alkaline phosphatase (IAP) detoxifies bacterial lipopolysaccharides (LPS; endotoxins) by dephosphorylation. Butyrate, a short-chain fatty acid produced as a result of bacterial fermentation, stimulates IAP gene expression. IAP may also modulate composition of microbial community via regulation of secretory IgA release. Fecal calprotectin is a commonly used marker for increased neutrophil (NEU) migration and local inflammation in patients with gastrointestinal diseases. Lower luminal IAP-activity may lead to increased production of toxic LPS-molecules, which in turn may stimulate Factor XII regulated contact activation pathway in the gut. Subsequent activation of kallikrein-kinin system by endotoxins may potentiate leakiness of the intestinal wall. Modified from Lassenius et al. (103).

In relation to the manifestations of the gastrointestinal diseases, there are potential novel biological mechanisms associated with the activity and permeability of the microbial toxins in the gut. New evidence supports the hypothesis that intestinal alkaline phosphatase (IAP) and components of the kallikrein-kinin system play an important role in the detoxification and the transport of bacterial endotoxins (Figure 2).

### Intestinal alkaline phosphatase

The human gut contains about 1.0–1.5 kg of bacteria. Notably, the brush-border enzyme intestinal alkaline phosphatase (IAP) plays an important role in the gut mucosal defense by suppressing inflammatory mediators, including microbial compounds (endotoxins, polyphosphates), and luminal adenosine triphosphate by dephosphorylation. IAP has also been shown to be involved in the regulation of duodenal bicarbonate secretion and fatty acid absorption. A decrease in the luminal IAP activity could increase the risk of gastrointestinal disease through changes in the microbial composition, intestinal inflammation, and the gut permeability (104, 105). Exogenous administration of IAP has on the other hand been shown to have potential therapeutic effects. In animal models of intestinal damage, oral supplementation of IAP reduced the gut epithelial damage and inflammation (106). Moreover, administration of exogenous IAP to mice suppressed the Salmonella and Clostridium associated disease activity and improved survival (107, 108). Interestingly, in IAP-knockout mice, the development of the metabolic syndrome was also prevented by oral IAP supplementation (109, 110). Both recombinant and bovine IAP has been utilized in human clinical trials. Promising results has so far been obtained from the treatment of patients with rheumatoid arthritis, heart surgery, sepsis, acute kidney injury, and inflammatory bowel disease (110–112). The histone deacetylase inhibitor butyrate, produced by intestinal bacteria through carbohydrate fermentation, is a positive activator of IAP gene expression. Oral butyrate supplementation has also shown beneficial effects in individuals with various gastrointestinal problems such as irritable bowel syndrome, inflammatory bowel disease, diverticulitis, and traveler's diarrhea (113). Butyrate has also been shown to alleviate renal fibrosis, inflammation, and kidney damage in rodent animal models (114–116).

Importantly, we recently observed in individuals with type 1 diabetes that decreased fecal IAP activity was accompanied by lower fecal butyrate, and IgA antibody concentrations. Of note, individuals with uncomplicated type 1 diabetes also exhibited higher fecal calprotectin concentrations compared to the non-diabetic controls. It was also shown that oral IAP supplementation increased the mucosal IgA secretion, which modulate immunity and host-microbe interactions in the gut (103, 117). Reduced fecal IAP levels have been reported in individuals with inflammatory bowel disease, celiac disease, and type 2 diabetes (118–120). Hypothetically, low intestinal IAP-activity could result in increased translocation of cytotoxic bacterial compounds, higher systemic endotoxin activity, and could thereby increase the risk of kidney or other organ injuries. In IAP-knockout mice, the dextran sodium sulfate-induced colitis phenotype was significantly aggravated compared to dextran sodium sulfate-treated wild-type mice. Oral administration of calf IAP attenuated the intestinal inflammation and normalized the mucosal architecture in dextran sodium sulfate-treated IAP-knockout mice (121). The first compound heterozygous mutations in the human *ALPI* gene leading to IAP deficiency have recently been described. Two index cases with different IAP mutations exhibited gastrointestinal manifestations similar to inflammatory bowel disease (122). A novel deletion-insertion mutation in the C-terminal end of the *hALPI* gene, was recently associated with familial hyperphosphatemia in Japan (123).

The effects of exogenous IAP administration have been evaluated in recent human clinical trials involving patients with sepsis, heart surgery, and rheumatoid arthritis. Of note, intravenously administrated bovine IAP has been shown to have promising renoprotective effects in sepsis-induced acute kidney injury (124–126). In animal models of intestinal damage, oral administration of IAP reduced gut epithelial damage, and inflammation (106, 127, 128). Despite these promising effects in animals, human trials with oral IAP supplementation are currently at the very early stage (111). However, endogenous IAP expression could potentially be enhanced via modulation of the gut microbiome (diet, probiotics), dietary supplements (e.g., resistant starch, fiber, butyrate) or with specific drug-therapy (e.g., Rifampicin, Zanamivir) (108, 129, 130).

### Contact activation pathway

Patients with impaired intestinal barrier function also display anomalous changes in the factor XII (FXII; Hageman factor) regulated contact activation pathway. Upon contact with negatively charged surfaces and anionic compounds (e.g., glass, heparin, polyphosphates, dextran sulfate, endotoxins) FXII is autoactivated, which eventually leads to sequential proteolytic cleavage of the down-stream targets. Cleavage of high-molecular-weight kininogen leads to subsequent production of bradykinin, a potent vasodilator of blood vessels (131). The question arises whether bradykinin release could also be linked to the increased gut permeability e.g., bacterial endotoxins, which may in turn boost the activation of FXII in the submucosal tissue. The increased gut permeability and translocation of bacterial components may also aggravate systemic inflammation and renal damage.

Hereditary angioedema is a rare genetic defect, primarily caused by mutations in the *SERPING1* gene, associated with complement 1 inhibitor deficiency (132). Gene mutations associated with hereditary angioedema have also been found in the *Factor XII* and *plasminogen* genes (133–136). Moreover, the non-genetic and non-allergic form of angioedema has frequently been seen in subjects using angiotensin-converting enzyme inhibitors (132). Angioedema is commonly characterized by short-lived episodes of serious edema involving the lungs, the skin, and the gastrointestinal tract (137, 138). Over 90% of hereditary angioedema patients have experienced episodic or chronic abdominal pain, which is accompanied by other gastrointestinal symptoms such as diarrhea, nausea or vomiting (139). Similar complications have been reported in individuals with angiotensin-converting enzyme inhibitor-induced angioedema (140). Based on the surveillance of antihypertensive medication (angiotensin-converting enzyme inhibitors/angiotensin receptor blockers) in the UK between 2007 and 2014, gastroenteritis was shown to be a significant risk factor for acute kidney injury (141). In addition to the gastrointestinal problems, kidney related disorders have also been documented in patients with hereditary angioedema (142–148). Cutaneous and systemic lupus erythematosus are the most often described autoimmune conditions associated with hereditary angioedema. It has been estimated that ~20–25% of hereditary angioedema patients with a subsequent lupus diagnosis exhibit renal complications (149).

Defects in the contact activation pathway have also been reported in patients with inflammatory bowel disease. Zeitlin et al. reported significantly higher kinin-forming activity in the colonic muscle tissue isolated from patients with ulcerative colitis (150). In patients with inflammatory bowel disease, increased interstitial kallikrein activity has also been shown to correlate with the inflammation in the isolated tissue samples (151, 152). In an animal model of colitis, elevated levels of portal endotoxins were found in mice treated with dextran sodium sulfate (153). However, in wild-type mice with dextran sodium sulfate induced colitis, complement 1 inhibitor treatment reduced the severity of the disease (154). Furthermore, the use of bradykinin receptor blockers have been shown to improve the general health condition of mice with chemically induced colitis (155–157). Moreover, inhibition of the kallikrein-kinin system reduced peptidoglycan-polysaccharide induced enterocolitis also in rats (158–160). In support of these earlier findings, Wang et al. has recently demonstrated that mice deficient in kallikrein, kininogen, or bradykinin receptors are protected from dextran sodium sulfate-induced mucosal damage and inflammation (161). In addition to the dextran sodium sulfate-induced gut related phenotype, the presence of other extra-intestinal manifestations should also be considered. Noteworthy, dextran sodium sulfate-induced colitis has also been associated with the development of acute kidney injury in mice (162, 163). There is a substantial number of studies supporting the impact of the kallikrein-kinin system on the development of microvascular complications such as diabetic retinopathy, macular edema, and diabetic nephropathy in patients with diabetes (164, 165). Apparently, more studies are needed to elucidate whether the contact activation pathway play a significant role in the pathogenesis of gastrointestinal and kidney related disorders.

## Conclusion

Based on the present overview, interconnections between gastrointestinal and renal disorders exist. One of the future challenges is to find diagnostic tools to determine the incidence of gut related diseases especially among patients with type 1 diabetes. Early identification and effective management of gut related disorders may slow down or even prevent the development of secondary complications e.g., renal and vascular injuries. In the future novel treatment strategies could potentially be adapted once the pathophysiological mechanisms behind the gut-kidney axis has been clarified.

## Author contributions

ML reviewed the literature and wrote manuscript. P-HG reviewed and edited the manuscript. Both authors made intellectual contribution to the present work and approved the final version of the manuscript for publication.

### Conflict of interest statement

P-HG has received research grants from Eli Lilly and Roche, is an advisory board member for AbbVie, Astra Zeneca, Boehringer-Ingelheim, Cebix, Eli Lilly, Janssen, MSD, Medscape, Novartis, and Sanofi. He has received lecture fees from Astra Zeneca, Boehringer-Ingelheim, Eli Lilly, Elo Water, Genzyme, Medscape, MSD, Novartis, Novo Nordisk, and Sanofi. The remaining author declares that the research was conducted in the absence of any commercial or financial relationships that could be construed as a potential conflict of interest.
